# Immunological Changes in Peripheral Blood of Ankylosing Spondylitis Patients during Anti-TNF-*α* Therapy and Their Correlations with Treatment Outcomes

**DOI:** 10.1155/2021/1017938

**Published:** 2021-10-15

**Authors:** Rongjuan Chen, Hongyan Qian, Xiaoqing Yuan, Shiju Chen, Yuan Liu, Bin Wang, Guixiu Shi

**Affiliations:** Xiamen Key Laboratory of Rheumatology and Clinical Immunology, The First Affiliated Hospital of Xiamen University, Xiamen 361003, China

## Abstract

Tumor necrosis factor-*α* (TNF-*α*) inhibitors are the main types of biological conventional synthetic disease-modifying antirheumatic drugs and have efficacy in treating ankylosing spondylitis (AS) which is not sensitive for nonsteroidal anti-inflammatory drug. However, the impact of TNF-*α* inhibitors on immune cells in patients with AS is still clearly undefined, and the impact of immune cells on treatment response is also largely elusive. This study is aimed at evaluating the longitudinal changes of circulating immune cells after anti-TNF-*α* therapy and their associations with treatment response in AS patients. Thirty-five AS patients receiving the treatment of anti-TNF-*α* therapy were included into this prospective observational study. The frequencies of immune cells including Th1, Th2, Th17, regulatory T cell (Treg), T follicular helper cell (Tfh), and regulatory B cell (Breg) in the peripheral blood were measured by flow cytometry at baseline and 4 time points after therapy. The difference in the circulating immune cells between responders and nonresponders was compared. This study suggested that anti-TNF-*α* therapy could significantly reduce circulating proinflammatory immune cells such as Th17 and Tfh, but significantly increased the percentages of circulating Treg and Breg. Moreover, circulating Breg may be a promising predictor of response to anti-TNF-*α* therapy in AS patients.

## 1. Introduction

Ankylosing spondylitis (AS) is a chronic inflammatory rheumatic disease characterized by inflammatory back pain and progressive ankylosing in spine [1]. AS can result in impaired physical functions including disability and obviously reduced life quality [2, 3]. Nonsteroidal anti-inflammatory drug (NSAID) is the main recommended first-line drug for the treatment of AS [4]. However, NSAID is not effective for some AS patients especially for those with later stages, and a large part of AS patients are still poorly controlled in clinical practice [5]. Thus, those AS patients need additional treatment with conventional synthetic disease-modifying antirheumatic drugs (DMARDs) or biological DMARDs [5–7]. Tumor necrosis factor-*α* (TNF-*α*) inhibitors are the main types of biological DMARDs and have a well-established efficacy in treating AS, which has largely revolutionized the treatment of AS in the past two decades [8]. Nevertheless, the treatment response is various involving high risk of infections among some patients such as tuberculosis [9, 10]. Improvement of AS patients' personalized therapy strategy is an urgent need for the heterogeneity in both the pathogenesis and treatment outcomes [11, 12]. To improve treatment outcomes, minimize infection risk, and reduce costs, it is critical for clinicians to identify responders to specific biological DMARDs and make adequate therapeutic decisions.

The roles of T cell subsets in the pathogenesis of AS have been reported in plenty works [13–15], and Th17 cells play a critical pathogenic role in the development of AS [13, 16]. Apart from Th17, other T cell subsets such as Th1 [14, 17] and T follicular helper cell (Tfh) which is correlated with B cell subtypes [18–20] are also involved in the pathogenesis of AS. Besides, several studies confirm that B cells participate in the pathogenesis of AS, such as increasing regulatory B cell (Breg) in peripheral blood of AS [21–24]. Nevertheless, the impact of anti-TNF-*α* therapy on those immune cells in AS patients is still not clearly defined, and the impact of immune cells on treatment response is also largely elusive. To evaluate the longitudinal changes of circulating immune cells after anti-TNF-*α* therapy and their associations with treatment response in AS patients, we performed a prospective observational study of AS patients receiving anti-TNF-*α* therapy.

## 2. Methods

### 2.1. Study Design and Patients

Active AS patients aged 20-65 years were recruited in the department of rheumatology in The First Affiliated Hospital of Xiamen University. The patients were recruited prospectively and followed up to 6 months after beginning anti-TNF-*α* therapy. Inclusion criteria were as follows: (1) patients met the 1984 modified New York classification criteria for AS; (2) without treatment history of biological DMARDs such as anti-TNF agents, anti-IL-17 agents, and anti-IL-6 agents; (3) with a Bath Ankylosing Spondylitis Disease Activity Index (BASDAI) score of no less than 1; (4) data of clinical characteristics and laboratory testing analyzed in this study were available; (5) receiving a standard treatment of anti-TNF-*α* inhibitors; and (6) without obvious infections such as tuberculosis. Exclusion criteria were as follows: (1) AS patients had been treated with biologics such as anti-TNF drugs or anti-IL-6 drugs, (2) patients with a history of spinal or joint surgery, (3) patients with other serious diseases such as cancer or cardiovascular diseases, (4) patients had serious adverse events and discontinued treatment, and (5) data of clinical characteristics and laboratory testing analyzed in the present study were not recorded. A total of 35 AS patients meeting both the inclusion and exclusion criteria were finally included between September 2018 and January 2019. The study was approved by the ethics committee of our hospital, and written informed consent was obtained from included patients.

### 2.2. Outcome Assessment and Data Collection

The primary endpoint was to achieve an improvement of no less than 50% in patients at 6 months according to BASDAI. Patients received routine monitoring of disease activity at 5 treatment stages including baseline, 1 month, 2 months, 3 months, and 6 months. Patients with a BASDAI 50% improvement after 6-month treatment were defined as responders, while those failed to gain a BASDAI 50% improvement were defined as nonresponders. Other clinical and laboratory parameters such as disease duration, erythrocyte sedimentation rate (ESR), and C-reactive protein (CRP) were recorded prospectively at the follow-up visit.

### 2.3. Sample Collection and PBMC Isolation

Peripheral venous blood was collected from each patient at baseline (before treatment) and 4 follow-up stages after the initiation of anti-TNF treatment (1 month, 2 months, 3 months, and 6 months). Serum and plasma were collected for the measurement of liver and renal function parameters. 5 ml peripheral venous blood was used for PBMC isolation with Ficoll-Paque density gradient centrifugation. The isolated PBMCs were stored at −80°C until analysis.

### 2.4. Flow Cytometry Phenotype

The frequencies of immune cells including Th1, Th2, Th17, regulatory T cell (Treg), Tfh, and Breg in the peripheral blood were measured by flow cytometry. Briefly, PBMCs were isolated and incubated with PMA (10 ng/ml, eBioscience) and BFA (10 *μ*g/ml, eBioscience) for 4 h then harvested and washed twice for 30 min. Then, cells were stained with anti-CD4 and anti-CD25 for 30 min at 4°C. During the intracellular staining, antibodies against IFN-*γ*, IL-4, and IL-17A were according to stain Th1, Th2, and Th17, respectively. Intracellular FoxP3 was also stained, and CD4^+^FoxP3^+^ was used to determine Treg. CD19^+^CD24^High^CD38^High^ cells were determined as Breg. CD4^+^PD1^+^CXCR5^+^ cells were determined as Tfh. The following anti-human antibodies for surface staining or intracellular staining were used: PE-CY7-anti-CD4, PE-CY5.5-anti-CD25, FITC-anti-IL-17A, PE-anti-Foxp3, FITC-anti-IFN-*γ*, PE-anti-IL-4, FITC-anti-CD19, PE-anti-CD24, PE-CY7-anti-CD38, PE-anti-PD1, and FITC-anti-CXCR5 (all eBioscience).

### 2.5. Statistical Analysis

Continuous variables were presented as mean ± standard deviation (SD) or median with quartiles (Q25 − Q75). Difference between responders and nonresponders was determined using Student's *t*-test or Mann-Whitney *U* test. Difference for data at different time points was assessed with paired *t*-test. The roles of immune cells at baseline in predicting treatment response were assessed by receiver operating characteristic (ROC) analysis, and the area under the ROC curve (AUC) was calculated. Statistical analyses were performed with STATA (Version 12.0, StataCorps, Texas, USA). Two-sided *P* values less than 0.05 were considered statistically significant.

## 3. Results

### 3.1. Clinical Characteristics of AS Patients

Table 1 summarized the clinical and laboratory characteristics of those AS patients (Table 1). Among those 35 AS patients, 30 (85.7%) were males. The mean age was 33.1 ± 8.8 years old, and the mean disease duration was 8.7 ± 5.1 years. At baseline, the mean ASDAS-CRP and BASDAI were 2.8 ± 0.8 and 4.4 ± 1.0, respectively. After anti-TNF-*α* therapy of 6 months, both ESR and CRP were significantly reduced (*P* < 0.05; Table 1). The mean ASDAS-CRP significantly declined to 1.4 (*P* < 0.001) and BASDAI declined to 1.9 (*P* < 0.001) at 6 months. Based on BASDAI, the response rate at 6 months after anti-TNF-*α* therapy was 60.0% (21/35).

### 3.2. Changes of Circulating Immune Cells after Anti-TNF-*α* Therapy

**Figure 1:**
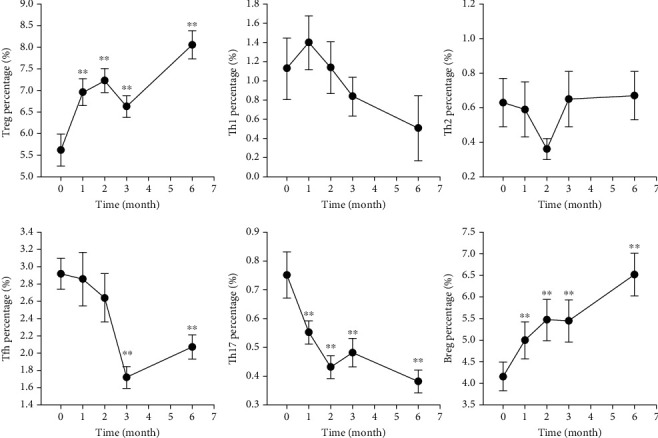
Changes of circulating immune cells after anti-TNF-*α* therapy in AS patients. The percentages of immune cells during follow-up were compared with that at baseline. ^∗^*P* < 0.05; ^∗∗^*P* < 0.005.

Th1, Th17, and Tfh are common proinflammatory immune cells. After anti-TNF-*α* therapy, both Th17 and Tfh decreased gradually, and there was also a modest but not significant reduction in Th1 (Figure 1). Anti-TNF-*α* therapy significantly reduced the percentage of circulating Th17 at 1 month after treatment (*P* < 0.005), and the effect was maintained through treatment course (Figure 1). Anti-TNF-*α* therapy began to significantly reduce the percentage of circulating Tfh at 3 months after treatment (*P* < 0.005), and the effect was also significantly lower at 6 months after treatment (*P* < 0.005). The mean percentage of circulating Th17 significantly decreased from 0.75 to 0.38 after 6 months of anti-TNF-*α* therapy (*P* < 0.001; Table 2).

Th2, Treg, and Breg are key immunoregulatory immune cells. After anti-TNF-*α* therapy, both Treg and Breg increased gradually, but the frequency of Th2 was not significantly changed (Figure 1). Anti-TNF-*α* therapy began to significantly increase the percentages of both Treg and Breg at 1 month after treatment (*P* < 0.005). The mean percentage of circulating Treg significantly increased from 5.62 to 8.06 after 6 months of anti-TNF-*α* therapy (*P* < 0.001), and the mean percentage of circulating Breg significantly increased from 4.16 to 6.52 (*P* < 0.001; Table 2).

### 3.3. Correlations of Circulating Immune Cells with Response to Anti-TNF-*α* Therapy in AS Patients

**Figure 2:**
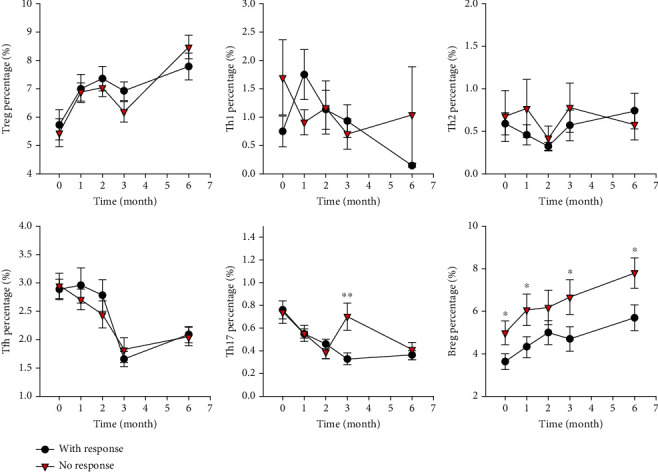
Changes of circulating immune cells after anti-TNF-*α* therapy in AS patients stratified by treatment response. Red triangle was for those responders, while black circle was for nonresponders. Difference between responders and nonresponder at each time point was compared. ^∗^*P* < 0.05; ^∗∗^*P* < 0.005.

Baseline disease characteristics such as age, disease duration, and ASDAS-CRP were comparable between responders and nonresponders (Table 3). Compared with nonresponders, responders had lower levels of ESR (*P* = 0.035) and CRP (*P* = 0.018) but had higher BASDAI (*P* = 0.042) (Table 3). Compared with those responders, nonresponders had a higher percentage of circulating Breg both at baseline and during follow-up (*P* < 0.05) (Figure 2 and Tables 3 and 4). There was no obvious difference in the baseline percentages of other immune cells such as Th17, Treg, and Tfh between nonresponders and responders (Table 3), and similar findings were also found at 6 months after anti-TNF-*α* therapy (Table 3).

**Figure 3:**
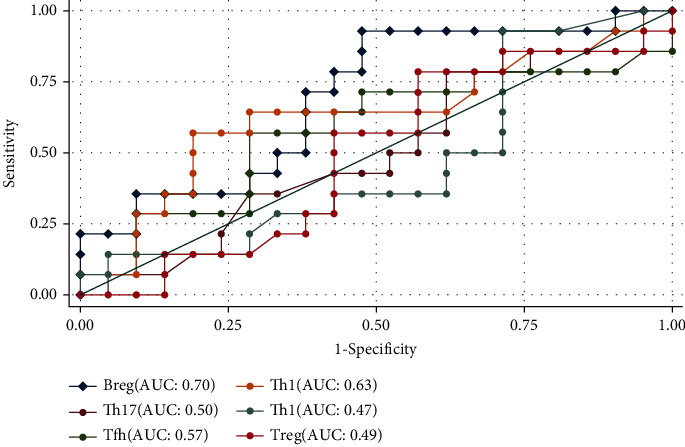
Assessment of the roles of circulating immune cells at baseline in predicting response to anti-TNF-*α* therapy in AS patients through ROC analysis (AUC: area under the ROC curve).

ROC analysis suggested that Breg was the best circulating cell in predicting response to anti-TNF-*α* therapy in AS patients (AUC = 0.70, 95% CI 0.52-0.88). Other immune cells had limited roles in predicting response to anti-TNF-*α* therapy (Figure 3).

## 4. Discussion

The impact of TNF-*α* inhibitors on immune cells in AS patients is still not clearly defined. Besides, the impact of immune cells on treatment response to TNF-*α* inhibitors is also largely elusive. This study was thus designed to prospectively evaluate the longitudinal changes of circulating immune cells after anti-TNF-*α* therapy and their associations with treatment response in AS patients. To our knowledge, this is the first prospective study investigating the impact of immune cells on treatment response to TNF-*α* inhibitors. We found that both Th17 and Tfh were reduced gradually by anti-TNF-*α* therapy, while Treg and Breg were increased gradually. Moreover, there was some immunological difference between treatment responders and nonresponders, and responders had a higher percentage of circulating Breg both at baseline and during follow-up, suggesting Breg as a possible predictor of response to anti-TNF-*α* therapy in AS patients.

AS is a heterogeneous disease, and it has been cleared that AS patients have various response to anti-TNF-*α* therapy [25–27]. BASDAI at baseline has an important impact on assessing the response to therapy in AS [28]. Our work showed that compared with nonresponders, responders had higher BASDAI scores and significant response to anti-TNF-*α* therapy. This study revealed that about 60% patients had at least 50% improvement in BASDAI at 6 months after anti-TNF-*α* therapy, while the others had poor response. Identification of predictors of treatment outcomes in AS patients is critical for clinicians to make adequate therapeutic decisions and provide personalized therapy for AS patients [5–27, 29–31]. Currently, there is still lack of definite predictors of response to anti-TNF-*α* therapy in AS patients. A recent systematic review and meta-analysis revealed that several clinical factors such as young age, male sex, and baseline BASDAI were predictors of better response to anti-TNF-*α* therapy in AS patients [28]. Serological markers such as baseline CRP and HLA-B27 were also identified as predictors of response to anti-TNF-*α* therapy in AS patients [28]. Nevertheless, the personalized therapy for AS patients is still difficult owing to the limited evidence from clinical studies or the lack of effective predictors [27, 32]. In this study, we assessed the roles of peripheral immunological profiles such as Th17, Treg, and Breg in predicting response to anti-TNF-*α* therapy of AS patients which are all HLA-B27 positive. We found that Breg was a possible predictor of response to anti-TNF-*α* therapy in AS patients, but the other immune cells such as Th17, Tfh, and Treg were not candidate predictor of response to anti-TNF-*α* therapy. The findings may be helpful to identify predictors of response to anti-TNF-*α* therapy and improve personalized therapy for AS patients from the perspective of immunological profiles in peripheral blood.

A major finding in our study is the potential role of Breg as a predictor of response to anti-TNF-*α* therapy in AS patients. Though anti-TNF-*α* therapy could increase the percentage of circulating Breg in AS patients, treatment nonresponders had a higher percentage of circulating Breg both at baseline and during follow-up, suggesting Breg as a possible predictor of response to anti-TNF-*α* therapy (Figure 2 and Tables 3 and 4). Several studies had assessed the changes of B cells with a regulatory phenotype in AS patients [21, 33, 34]. Cantaert et al. firstly reported that spondylarthritis patients had increased circulating B cells with a regulatory phenotype (CD19^+^CD5^+^) [21]. A study by Bautista-Caro et al. also reported that AS patients had increased circulating CD19^+^CD24^hi^CD38^hi^ B cells with regulatory capacity, and anti-TNF-*α* therapy could significantly reduce the number of these B subset cells [34]. However, another study reported similar frequencies of CD24^+^CD38^+^ B cells between AS patients and controls, but those cells from AS patients produced less IL-10 and thus had functional defects [33]. To our knowledge, apart from those 3 studies, no other study on the roles of Breg in AS has been published. Our study revealed that Breg was possibly related to response to anti-TNF-*α* therapy in AS patients, and patients with high frequencies of Breg may predispose to poor response to anti-TNF-*α* therapy, which provides new insights into the roles of Breg in AS. Currently, the molecular mechanism underlying the roles of Breg in the pathogenesis of AS is still unclear and needs to be elucidated in future studies.

While there are emerging data providing evidence for the involvement of T cell subsets in the pathogenesis of AS, few studies have evaluated the longitudinal changes of circulating immune cells after anti-TNF-*α* therapy in detail [35–37]. Additionally, our knowledge about their roles in predicting response to anti-TNF-*α* therapy in AS patients is still limited. The findings from our study confirmed the reduction in the frequencies of circulating lymphocyte subsets after anti-TNF-*α* therapy in AS patients. This study suggested that anti-TNF-*α* therapy could significantly and selectively reduce circulating proinflammatory immune cells such as Th17 and Tfh, but significantly increased the percentage of circulating Treg. While a large part of AS patients had gradual reductions in the percentage of CD4^+^ subsets such as Th17 and Tfh, some patients had increased percentages of circulating Th17 or Tfh after anti-TNF-*α* therapy, indicating the existence of variability in treatment response among AS patients. In addition, none of those T cell subsets were obviously related to response to anti-TNF-*α* therapy in AS patients (Figure 2 and Tables 3 and 4), suggesting that T cell subsets may have limited roles in predicting response to anti-TNF-*α* therapy in AS patients.

Our study suggested that anti-TNF-*α* therapy began to significantly increase the percentage of circulating Treg at 1 month after treatment (*P* < 0.005), and its mean percentage significantly increased from 5.62 to 8.06 after 6 months of anti-TNF-*α* therapy (*P* < 0.001, Table 2). It is uncertain whether the increase of Treg after anti-TNF therapy is responsible in part for the benefit of anti-TNF therapy in treating AS. A recent study suggested that expanding Treg through low-dose IL-2 was effective in treating AS [38]. The effect of anti-TNF-*α* therapy in treating AS may be at least partially mediated by its roles of increasing Treg cells, which need to be further studied.

This study used a prospective design and thus could provide a better assessment of the immunological changes in peripheral blood during anti-TNF-*α* therapy than those studies using retrospectively collected data. However, the findings in our study should be interpreted with caution because the sample size was not large enough. Besides, the treatment duration in this study was 6 months, which could not evaluate either the long-term efficacy of anti-TNF-*α* therapy or the long-term impact of anti-TNF-*α* therapy on immune cells. Further studies with larger number of AS patients and long-term follow-up are recommended to provide more evidence.

In summary, this study suggested that anti-TNF-*α* therapy could significantly reduce circulating proinflammatory immune cells such as Th17 and Tfh, but significantly increased the percentages of circulating Treg and Breg in AS patients. Moreover, circulating Breg may be a promising predictor of response to anti-TNF-*α* therapy in AS patients. Further prospective cohort studies with larger number of AS patients and long-term follow-up are warranted, and the molecular mechanism underlying the roles of Breg in the pathogenesis of AS needs to be elucidated.

## Figures and Tables

**Table 1:** Clinical characteristics of total 35 patients at baseline and after follow-up.

Characteristic	At baseline	6 months	*P* value
ESR (median[Q25-Q75])	21 (9-34)	4 (2-11)	<0.001
CRP (median[Q25-Q75])	6.2 (1.9-21.3)	1.6 (0.5-3.9)	0.002
ASDAS-CRP	2.8 ± 0.8	1.4 ± 0.8	<0.001
BASDAI	4.4 ± 1.0	1.9 ± 1.3	<0.001

AS: ankylosing spondylitis; data were shown as mean ± SD or median [Q25 − Q75].

**Table 2:** Changes of immune cells among total 35 patients during follow-up.

Immune cells	0 month	6 months	*P* value
Treg	5.62 ± 2.19	8.06 ± 1.98	<0.001
Th17	0.75 ± 0.37	0.38 ± 0.18	<0.001
Th17/Treg	0.15 ± 0.10	0.05 ± 0.03	<0.001
Tfh	2.92 ± 0.80	2.07 ± 0.62	<0.001
Th1	0.30 (0.13-1.27)	0.18 (0.03-0.25)	0.003
Th2	0.38 (0.09-0.87)	0.29 (0.14-0.89)	0.71
Th1/Th2	1.55 (0.44-5.33)	0.28 (0.10-1.38)	<0.001
Breg	4.16 ± 1.94	6.52 ± 2.89	<0.001
CD3	73.23 ± 7.98	67.45 ± 10.15	0.010
CD4	38.04 ± 5.31	32.25 ± 7.64	<0.001
CD8	28.49 ± 8.44	26.22 ± 8.62	0.27
CD4/CD8	1.47 ± 0.51	1.38 ± 0.57	0.48

Data were shown as mean ± SD [standard deviation] or median [Q25 − Q75].

**Table 3:** Differences in baseline clinical characteristics and immune cells between responders and nonresponders.

Items	Responders (*N* = 21)	Nonresponders (*N* = 14)	*P* value
Gender (male, %)	18 (85.7%)	12 (85.7%)	1.00
Age (year, mean ± SD)	32.86 ± 7.53	33.43 ± 10.73	0.85
Disease duration (year, mean ± SD)	8.10 ± 5.21	9.71 ± 5.07	0.37
ESR (median[Q25-Q75])	13 (6-30)	29 (14-49)	0.035
CRP (median[Q25-Q75])	2.70 (1.19-15.72)	8.50 (6.65-33.63)	0.018
ASDAS-CRP	2.73 ± 0.72	2.98 ± 0.84	0.35
BASDAI	4.68 ± 0.87	3.99 ± 1.05	0.042
Treg	5.73 ± 2.44	5.45 ± 1.83	0.712
Th17	0.76 ± 0.38	0.74 ± 0.36	0.880
Th17/Treg	0.16 ± 0.10	0.15 ± 0.11	0.948
Tfh	2.89 ± 0.81	2.95 ± 0.81	0.816
Th1	0.18 (0.13-0.64)	0.81 (0.15-2.62)	0.200
Th2	0.43 (0.05-0.96)	0.28 (0.11-0.74)	0.749
Th1/Th2	0.52 (0.30-4.26)	2.30 (0.71-9.81)	0.178
Breg	3.62 ± 1.70	4.97 ± 2.05	0.041
CD3	73.62 ± 7.87	72.64 ± 8.40	0.729
CD4	38.02 ± 5.41	38.08 ± 5.36	0.976
CD8	28.26 ± 8.07	28.83 ± 9.27	0.849
CD4/CD8	1.48 ± 0.54	1.45 ± 0.46	0.878

Data were shown as mean ± SD (standard deviation) or median [Q25 − Q75].

**Table 4:** Differences in immune cells at 6 months between responders and nonresponders.

Immune cells	Responders (*N* = 21)	Nonresponders (*N* = 14)	*P* value
Treg	7.79 ± 2.22	8.47 ± 1.52	0.323
Th17	0.36 ± 0.16	0.41 ± 0.21	0.356
Th17/Treg	0.05 ± 0.03	0.05 ± 0.02	0.838
Tfh	2.08 ± 0.65	2.06 ± 0.59	0.917
Th1	0.14 (0.03-0.24)	0.20 (0.05-0.30)	0.204
Th2	0.29 (0.12-1.29)	0.25 (0.17-0.77)	0.590
Th1/Th2	0.25 (0.09-1.19)	1.18 (1.10-1.50)	0.449
Breg	5.68 ± 2.79	7.78 ± 2.64	0.033
CD3	68.02 ± 9.50	66.60 ± 11.37	0.690
CD4	32.71 ± 7.03	31.55 ± 8.71	0.666
CD8	25.76 ± 7.62	26.92 ± 10.20	0.703
CD4/CD8	1.40 ± 0.56	1.35 ± 0.60	0.799

Data were shown as mean ± SD (standard deviation) or median [Q25 − Q75].

## Data Availability

All data are available upon request.
